# Primary Care Wound Clinics: A Qualitative Descriptive Study of Patient Experiences in Community Pharmacies

**DOI:** 10.3390/pharmacy10040099

**Published:** 2022-08-17

**Authors:** Lusi Sheehan, Sheldon Dias, Michael Joseph, Sahil Mungroo, Jake Pantinople, Kenneth Lee

**Affiliations:** 1 Lusi Sheehan, Pharmacy 777, Applecross, WA 6153, Australia; 2 Sheldon Dias, Pharmacy 777, Bridgetown, WA 6255, Australia; 3 School of Population and Global Health, The University of Western Australia, Crawley, WA 6009, Australia; 4 Jake Pantinople, Pharmacy 777, Karratha, WA 6714, Australia; 5 School of Allied Health, The University of Western Australia, Crawley, WA 6009, Australia

**Keywords:** acute wound, patient experience, pharmacy, primary care, qualitative, wound care

## Abstract

The expansion of primary care wound services serves to alleviate secondary and tertiary care utilization. However, patient satisfaction is required to ensure service uptake. In recent years, various community pharmacies in Australia have begun to offer dedicated wound clinics; however, evaluations of patient experiences have yet to be conducted. Thus, the present study seeks to explore: (1) the experiences and satisfaction of patients who have received wound care consultations for their acute wounds in a community pharmacy setting; and (2) how current pharmacy-based wound services can be improved. Semi-structured individual interviews were conducted with patients across five pharmacy-based wound care clinics in Western Australia. Interviews were audio-recorded, transcribed verbatim, and imported into QSR NVivo 12 Plus. Interview transcripts were coded and thematically analyzed using the framework method. Twelve interviews were required to reach data saturation. Five key themes emerged: the accessibility of wound services, the comprehensiveness of wound care services, confidence in wound care consultants, the awareness and promotion of wound services, and the expansion of wound care services. Overall, participants were satisfied with the accessibility and comprehensiveness of pharmacy-based wound service delivery, trusted the health care providers, and wanted the service to be expanded. The reported patient satisfaction, confidence in the health care provider, and desire to expand the service suggests there is potential for the service to grow in Australia. Due to the growing costs of wound care globally, there is scope to further evaluate and expand wound care services in the primary care setting on an international level.

## 1. Introduction

Wound care is an essential aspect of health care and comprises a significant proportion of health expenditure globally [1]. For example, in Australia, wound care services comprised 2% of total health spending in 2019 (over AUD 3.5 billion) [1]. Globally, the annual wound care cost was an average of AUD 2.6 billion in 2014, and this was predicted to rise to AUD 3.5 billion in 2021 [2]. Economically, the annual wound care products market is predicted to reach AUD 15–22 billion by 2024 [2]. As such, it is important to optimize the provision of essential services such as wound care in primary care sectors to alleviate higher costs in secondary and tertiary care settings.

In Australia, wound care services in the primary care sector are provided by community pharmacies, General Practices (GPs), and nurse-led clinics [3,4]. While the management of acute wounds is within the scope of practice of all registered pharmacists in Australia [5], dedicated wound clinic services, provided by pharmacists within community pharmacies, have only commenced in recent years. To date, patients’ perceptions and experiences of wound treatment in GP clinics and nurse-led clinics have been explored [1,3,6,7], and extant literature suggest a level of patient dissatisfaction with wound care in the GP clinic setting, with issues cited including time constraints and the level of care provided [7,8,9]. In contrast, patients’ perceptions and experience in the pharmacy setting have yet to be explored.

Given that pharmacists are readily accessible health professionals and appointments are typically not required, community pharmacies have the potential to further expand access to primary care wound services. However, to ensure patients are willing to access care from pharmacists, it is important to firstly understand patients’ current experiences and satisfaction of wound care services in the pharmacy setting. As such, the present study aims to explore:

1. The experiences and satisfaction of patients who have received wound care consultations for their acute wounds from a pharmacy-based wound care clinic;
2. Patients’ perceptions on how current pharmacy-based wound care services can be improved.

## 2. Materials and Methods

### 2.1. Study Design

The present study utilized a qualitative descriptive design, and data were collected via semi-structured individual interviews. This style of interview permits flexible data collection and promotes rich accounts for data analysis [10].

### 2.2. Ethical Statement

Ethics approval was granted by the University of Western Australia Human Research Ethics Office (2021/ET000148). Blinding was not conducted as it was not appropriate for the given study design; however, participant names were not included in the analysis and the present study report to ensure anonymity.

### 2.3. Participants and Recruitment

Participants were purposively recruited by four members of the research team between July and August 2021.

At the time of recruitment, one of the study authors was an employee of one of the recruitment sites (pharmacy-based wound care clinic) and was consequently excluded from the recruitment process due to potential conflicts of interest. While there are no restrictions for community pharmacies to offer wound care services at present, all pharmacists within the recruitment sites chosen for the present study were chosen because they had received at least one wound care workshop delivered by a credentialed wound care clinician (as credentialed by Wounds Australia) with postgraduate qualifications in wound care; this served to ensure a level of consistency in service delivery, thereby facilitating the evaluation of the service.

Participants were included in the study if they met the following inclusion criteria:

1. Had received at least one wound care consultation in a pharmacy-based wound care clinic for an acute wound (defined as occurring within the last 3 months) [11];
2. Able to provide informed consent (i.e., able to converse and understand written and verbal English);
3. At least 18 years of age.

Recruitment continued until data saturation occurred, which was defined as the point where no new themes or sub-themes arose from patient interviews (via team consensus). However, an initial target of 15 participants was suggested based on guidelines for sample sizes in qualitative research [12]. Participants were recruited from five pharmacy-based wound care clinics in Western Australia. Participants were reimbursed for their time and travel (to the pharmacy) with an AUD 30 electronic gift card.

### 2.4. Interviews

Interviewers all received face-to-face qualitative interview training and underwent appropriate simulation activities with the senior author of the team (who has expertise in qualitative research) prior to the commencement of interviews. The interview guide was designed by the senior author in collaboration with another study author who is also a credentialed wound care clinician. The interview guide consisted of topics associated with participants’ wound care experiences and potential improvements to the service. Each interview was audio-recorded before being transcribed verbatim into Microsoft Word. Participants were interviewed face-to-face in participating pharmacies’ consultation rooms or via a Zoom teleconference between July and August 2021.

### 2.5. Data Analysis

Qualitative thematic analysis was conducted following the framework method [13]. Interview transcripts were imported into QSR NVivo 12 Plus for the coding and analysis of emergent themes. After familiarization with the interviews, the four interviewers created a working analytical framework in consultation with the senior author. The first transcript was then independently coded by five of the authors (including the senior author) using the working analytical framework, and codes were then compared and discussed to further refine the working analytical framework. Subsequent transcripts were independently coded by the interviewers, and the codes were reviewed as a team through a process of analyst triangulation to establish credibility and confirmability [14]. New codes were continuously reviewed by the research team before being incorporated into the working analytical framework. All decisions made around coding and thematic analysis were documented in weekly meetings to serve as an audit trail, thereby further establishing confirmability and enabling the potential for an inquiry audit to establish the dependability of the findings [14].

Codes were then charted into a framework matrix, whereby themes and sub-themes were identified (Table 1). These themes and sub-themes were presented to an audience comprising pharmacy academics and pharmacy students as a means of peer debriefing to further establish the credibility of the findings [14]. Negative cases are identified, discussed, and presented in the Results section (where relevant) to further establish credibility [14]. Finally, detailed participant quotes are presented and described to enable the establishment of the transferability of the findings [14].

## 3. Results

Data saturation was achieved by the 12th interview, and thus, recruitment was concluded at the 12th participant. These participants were recruited from one of five metropolitan-based community pharmacies, with eight participants being recruited from a single site, and one participant from each of the remaining four sites.

Participants ranged from 35 to 89 years old (median age 63.5 years), with 58% of participants being female. Patients had one or more wounds and made single or multiple visits to the pharmacy for wound treatment. Themes and sub-themes pertaining to the three domains of satisfaction, experiences, and improvements are shown below (Table 1).

### 3.1. Accessibility of Wound Care Services

#### 3.1.1. Timely Wound Care Services

Almost all participants mentioned time as a consideration for how accessible wound care services were to them. As illustrated in the first example in Table 1, it was suggested that shorter waiting times at pharmacies made them a more attractive wound care prospect than GP clinics.

Timeliness also encompassed wound care sessions which were long enough to provide more thorough care. Multiple participants also suggested that pharmacy-based wound care clinics offered more productive consultations because there was less pressure to finish quickly. This extra time was used to provide the patient with more extensive education on their wound:

*[previous non-pharmacy service] …It was okay, but it was rushed, because it was, you were really pressured for time, so I felt I didn’t have to time to ask any questions about the wound and when I came here, because they want to make sure they did a proper job. That gave me that extra maybe five minutes to actually discuss what the wound look like.*

[P10, female, aged 60]

#### 3.1.2. Flexibility of Wound Care Services

A notable influence on the accessibility of wound care services was the degree to which patients were able to organize their consultations around their daily schedules. Participants who attended pharmacies with a broader range of booking times were able to schedule appointments which were convenient to them. Most of the participants discussed how flexibility impacted their ease of access to these services. It was important for the participants to be able to match their availability with that of the pharmacy:

*Oh, that I liked. So specifically, it was accessible. Yeah, it was. There was like an easy to make a booking like imagine the day was incredibly easy for like … to say I’ll just see you on a Wednesday, she’s like these are my availabilities and I could then match it with my availability, it was seemed to be quite flexible.*

[P1, female, aged 41]

This was contrasted with the flexibility offered by other wound care providers:

*Well, this is really convenient to maybe come down here. Where going to the dermatologist is easy enough too but it’s actually awkward to bring up an appointment, you can’t only get an appointment up there really, unless the nurse is quite free. So having an appointment is easier, when you come down with pharmacists, because there’s more, there’s more opportunities. So that makes it easier.*

[P4, female, aged 60]

#### 3.1.3. Location of Wound Care Services

The proximity of the pharmacy to participants’ homes affected their ability to access that pharmacy’s wound care services. Over half of the participants suggested that the distance they had to travel contributed to their decision to receive wound treatment at specific pharmacies. Proximity to home was found to be especially important for participants whose previous wound care services had been far away:

*And the fact that I didn’t have to drive over an hour to the doctor’s surgery and get the dressings—even though I had to still go twice a week anyway … I like that it was close, able to book in at easier times.*

[P9, female, aged 35]

#### 3.1.4. Affordability

The cost of the service was a major consideration for participants in determining whether they could receive wound care at a pharmacy. Almost half of the participants stated that pricing was important in their decision to receive pharmacy-based wound care. Participants had contrasting views of the affordability of pharmacy-based wound care and made comparisons with other wound care providers such as GPs:

*It’s relatively cheap, you know, in terms of, instead of going to a GP, which is a bit that’s a big draw card for other people which is the affordability, the service was friendly.’*

[P1, female, aged 41]

*And the products are expensive, they’re 90 cents to swab, but they’re efficient. I rather have efficient than a scar.*

[P12, female, aged 55]

### 3.2. Comprehensiveness of Wound Care Services

#### 3.2.1. Adequate Facilities and Equipment

Most participants discussed whether they felt that the pharmacy had the equipment and spaces available to appropriately treat their wounds. The majority of the participants felt that adequate resources were essential to a comprehensive wound care service. One participant explained how their pharmacy had the appropriate resources to deal with the specifics of their wound and conceal its presence:

*…And it’s the same from this time when I have something, I’ve had something burnt on my face, and I couldn’t find any other right size bandages, they were all too big and wide. And they were obvious. So this is sort of a little more subtle, a new colour on the face. And so I feel more comfortable wearing it out and about. So yeah they’ve got the gear and the supplies for what I wanted.*

[P4, female, aged 60]

However, there are limitations to the wounds that pharmacies could treat due to not having the necessary equipment or expertise:

*Well, if you have a sprained ankle, you can’t expect a pharmacist to have an x-ray machine. I mean it’s fairly obvious that pharmacists are not hospital—don’t have the equipment that you can only get in a hospital and if the wound is severe, they might be able to apply let’s say a tourniquet or something and call an ambulance, but they obviously cannot perform things for which they’re not equipped.*

[P7, male, aged 89]

#### 3.2.2. Attention to Detail

The attentiveness of the wound care consultants forms an essential component of comprehensive wound care service. Multiple participants described how the pharmacist’s attention to detail led to a more thorough wound care service. Attention to detail allowed the pharmacist to promote patient-centered care. Pharmacists considered the finer details of a patient’s circumstances when determining what treatment was relevant to them. The attention to detail shown by pharmacists was seen as a way of avoiding complications in the wound treatment process:

*And the focus is what they do, whereas other medical professionals, provide a whole range of different services whereas this is focused on wound care for this. And also, the confidence knowing the person seeing so much on them when something is actually wrong to say no you’ve got a problem with this and a lot of people often traditionally other doctors for skin cancer or whatever will just give you the treatment and tell you to get on with it and I think that’s when problems can arise.*

[P3, male, aged 67]

In one case, a pharmacist was able to tailor their treatment plan based on the patient’s allergies:

*So I was helped by getting the proper care by someone who understood what was happening. Understood, going by my allergies that was really known by the pharmacist, so they knew what I could and could not tolerate on my skin, so therefore, the dressings, I got here the pharmacy … is perfect.*

[P10, female, aged 60]

#### 3.2.3. Wound Care Education

Receiving information on how one’s wound is progressing and how to self-manage was something which participants felt made the service more complete. Many participants commented on the education they had received during their consultations. However, one participant felt that the pharmacist could provide more education on their wounds because they had to prompt them for a response:

*Probably they could describe it a little bit more, but because I was keen to learn I was sort of asking them what the rationale was, and what they were doing. They probably just did the dressing without explaining as much. If I hadn’t have asked the question, I might not have learned so much. Perhaps a little bit more education would be useful in that process.*

[P4, female, aged 60]

Patients came to pharmacies to get advice on how they could manage wounds which were too severe to self-treat:

*I came here to get clarification, as what I said before, because the wound was quite deep and basically that was all I was looking for.*

[P5, male, aged 57]

There was also a sense that pharmacists had extensive wound care knowledge and shared their expertise with the participants:

*She also had her own ideas and concepts of dressings and stuff that I could use … She also knew about wounds as well. So she was able to give me her info which pretty much lined up with what the doctor had said.*

[P9, female, aged 35]

Often, pharmacists clarified information that the participants had received from previous consults:

*And the same thing, it was just sort of seeing it and low grade, a little bit of slough on it and I didn’t really, I thought I probably should use the same techniques as I’ve learned from here, but I wasn’t sure enough to do it. So yesterday I came down and one of the other pharmacists just basically says yeah you need to do the same thing, you need to keep it moist and keep it dressed. So that’s what I’m going to do now for a few weeks and see. Hopefully I won’t end up with a big scar.*

[P4, female, aged 60]

#### 3.2.4. Continuity of Care

Participants described how adequate follow-up appointments and ongoing education were critical to a thorough wound care service. Almost half of the participants identified examples of ongoing care they had received. Pharmacists would organize regular appointments so that the wounds could be continually managed:

*[Participant addresses coming in for wound care] ’And then you know I mainly come in every second, second or third day. Yeah, might have been every second day for a little bit and then every third day or something. Yep and then once a week, just so she could keep an eye on it and change the dressing you know and all that, I mean I probably could have done it with them [doctors] but it’s just as easy to come here and especially because it’s so it’s so close for me to come here so you know like I didn’t It’s not wasn’t an effort really.*

[P1, female, aged 41]

This after-care was an essential part of the wound care service and helped to reassure the participants as the treatment progressed:

*Yeah, you do need to provide some sort of after-care once you’re done [with the wound care service] And be worried about it. Then you need to reassure them that it’s okay. That it’s part of the procedure … It’s part of getting better.*

[P4, female, aged 60]

One participant was very impressed when the pharmacist contacted him weeks after his wound care to check on his progress:

*I was home about a fortnight and out of the blue she rang me. So she rang me and said “Oh I’m …. I was the lady that dressed your wound at the pharmacy, and I’m just ringing to see that you are alright. Is everything all right?”… So you meet all sorts of different people in life but this is the first time I experienced the care and attention of …, who after a fortnight, was being so thoughtful, and, and caring enough to ring me to ask me how I was going. I was impressed I gotta tell you.*

[P11, male, aged 78]

### 3.3. Confidence in Wound Care Consultants

#### 3.3.1. Adequate Wound Care Qualifications

Participants who knew that their wound care pharmacists were qualified to provide them with treatment felt confident that they would have good outcomes. The majority of participants alluded to the qualifications or training which the pharmacists held. There was an expectation that pharmacists had adequate training in wound care prior to conducting consultations:

*I think that it needs someone who has contemporary medical training to decide exactly what wounds can be treated locally by the pharmacist and what wounds would need someone with more specialized equipment perhaps and possibly more specialized knowledge.*

[P7, male, aged 89]

It was argued that adequate qualifications were essential due to the plethora of wound care scenarios one can encounter:

*These days, and the multiplicity of things that you need to know and remember when you look at the wound, whether it’s infected whether it’s swollen, whether it’s hot, cold. I think you’re better off to go to someone who’s had specialty training which … has had and I’m aware of that.*

[P2, male, aged 78]

#### 3.3.2. Wound Care Competency

Multiple participants discussed competency during the interviews. Participants felt confident when they got the sense that their wound care pharmacist was capable of managing their wounds. Competency entailed demonstrating an ability to provide a high standard of wound care through treatment and education. It is important to distinguish this from participants knowing that the pharmacist was accredited by Wounds Australia to provide specialized wound care. Only one participant mentioned the latter [P2]. Another participant described how they could not self-treat their wounds because they did not have the level of competency that the pharmacist did:

*I can’t manage this particular place [wound location]. I haven’t got the dressings in my first aid kit and if I came down here, they have the dressings, they have the skills and that was what I was feeling.*

[P8, female, aged 87]

Competency inspired confidence in participants and made them feel at ease throughout the wound care process:

*The attention to detail and making sure the wound was cleaned properly using a sterile tray putting down plastic and taking a lot of care and cleaning the wounds and making… measuring it. I just felt completely at rest and confident with the competency of the operator was going to give me a good result.*

[P2, male, aged 78]

#### 3.3.3. Professionalism of Wound Care Service

A few participants described how pharmacists conducted themselves in a professional manner throughout the course of the wound care—showing empathy and placing the greatest importance on the patient’s wellbeing. Some pharmacists displayed an air of professionalism throughout the whole consultation:

*The fact that I got a positive outcome and that the consultation I got with such expertise that was available to me locally, walk-in type arrangement, very convenient, obviously felt like I was dealing with a professional, so I had some degree of confidence with the information that was being given.*

[P5, male, aged 57]

Another participant appreciated that the wound care pharmacist was able to determine what needed to be done from the initial presentation of the wound, onwards:

*I liked the professionalism with regards to understanding exactly what the situation was in the beginning, and then monitor, I think online records can show pretty nasty, photos of the wound through progression from time I first came in to the end.*

[P3, male, aged 67]

### 3.4. Awareness and Promotion of Wound Care Services

Most participants were initially unaware of pharmacy-based wound care services until they were informed of them by pharmacists and other health professionals. Participants felt that this lack of awareness was also present in the wider public:

*I don’t think the public associate wound care, necessarily wound care dressing with pharmacy. I think they’re still a little bit naive about what it really entails. So something could be done to inform the public that all wounds aren’t the same.*

[P2, male, aged 78]

Consequently, participants argued that awareness of these wound care services need to be raised. Many participants suggested advertising was a means of promoting wound care (since they had found about the service in this way). Signs and word-of-mouth were among the suggestions for forms of advertisement:

*I think maybe I would of obviously never have known about it [pharmacy-based wound care service] unless I went in there and was asking about the bandaging and she [pharmacist] offered that she could help me with it, cause they offer that service. The price was good, so maybe it needs to be a little bit more advertised or something. Cause when I told people that I had it done they were like—what?! Your chemist does that? Even people I you know talk to had no idea that the chemist offer that.*

[P9, female, aged 35]

*Well, probably what you do, just the simple sign out the front will stimulate a conversation with people that come in and, you know, maybe a little bit detail about what sorts of wounds they can look after whether it’s ulcers or, you know, just sort of grazes or you know what level of care they could offer.*

[P4, female, aged 60]

### 3.5. Expansion of Wound Care Services

It was thought that expanding wound care will increase accessibility for a greater number of patients with wounds. Around half of the participants discussed the idea of expanding the service. Participants argued that expansion involved increasing the number of pharmacy-based wound care clinics and availability hours. One idea was to have a wound care pharmacist operating at every pharmacy:

*You know, I think, pharmacy generally in this space, in particular in … should go all out to make sure we’ve got a wound care pharmacist in each location and encourage people to use the service because it’s wonderful.*

[P2, male, aged 78]

Another participant argued that expansion would relieve the burden on doctors:

*[on expansion] I think they [pharmacists] might have a bit of trouble dealing with doctors on this because they don’t like other professionals to sort of encroach in their on their areas, but to be honest, they are, they [doctors] are incredibly busy too, and I don’t actually think they have the resources to manage all the different types of wound care that possibly could be done.*

[P4, female, aged 60]

## 4. Discussion

### 4.1. Principal Findings

This study explored patient experiences and satisfaction with pharmacists providing wound care consultations and ways these services could be improved. This study offered several insights into factors which influenced patient satisfaction, including the accessibility of service, comprehensiveness of service, and confidence in wound care consultants. It also highlighted how the awareness and promotion of wound care services could be improved.

Based on the findings of the present study, wound care which was timely, flexible, affordable, and located close to home improved accessibility for patients and led to greater satisfaction in the services. Examples of flexibility identified were walk-in access and convenient booking times. Prior research into wound care management (albeit chronic wounds in tertiary and primary care settings) also found that timeliness, location, and flexibility provided greater accessibility to wound care [1,8,15]. However, these same studies also reported dissatisfaction with prolonged waiting times, practitioner availability, and taking hours to organize transport [8,15]. Thus, the present study findings add to extant literature by suggesting that patient values around accessibility are supported in acute wound primary care settings.

The present study also highlights the importance of affordable services. There is a financial burden on patients when purchasing wound care products because these items are not currently remunerated [4,16]. Despite this financial burden and subsequent service costs, the present study found that most participants were reasonably satisfied with the current costs given the level of service, and this cost versus quality of wound care service consideration has been previously found [17]. Conversely, affordability issues are more prominent in chronic wound management [4]. Past research has noted that chronic wound costs have the potential to be at such a level that a patient’s relationships with family members became strained [4]. These contrasting experiences further outline the importance of including acute wound management in primary care settings, as this forms a service which people are willing to pay for and which can improve the quality of life of patients with wounds. Furthermore, appropriate acute care can limit the progression of wounds to chronicity and thereby avoid the financial burden of the subsequent treatment. The financial burden can be further limited by implementing chronic wounds into the service too.

When alluding to the comprehensiveness of service for acute wounds, adequate equipment and facilities, continuity of care, attention to detail, and wound care education were commonly found among participants within the present study. Although this was a prominent theme in this study, adequate wound care equipment has rarely been mentioned in prior studies. The lack of reimbursement associated with wound care products can steer wound management providers away from evidence-based treatments [4]. Given the importance of evidence-based protocols and products in wound management, patient dissatisfaction arises when wound clinics have inadequate equipment to manage their wounds [4].

Participant experiences with attention to detail and the pharmacy clinician’s perceived wound care competency were satisfactory for the patients in this study. Notably, patients appreciated the use of photography to map the progression of their wound. Utilizing imaging in the assessment and management of the wound helped established confidence in the wound care provider. Wound care providers’ abilities to demonstrate attention to detail and competency through the use of imaging have been referenced in various studies as contributing significantly to patient satisfaction [8,15,17,18]. However, competence should not only be measured by these two contributing factors, and further investigation is required to further probe patients’ views on the wound care providers’ competency.

Patients also heavily referenced wound care education and continuity of care. Wound care education was perceived as satisfactory by patients who came for clarification and treatment of their wounds. However, in previous studies, wound care education from clinicians is often deemed unsatisfactory, for example, in post-operative wound after-care [15]. Prior studies identify the desire for more education on specific wound types, how patients value timely education, how a lack of education can heighten anxiety, and how poor health literacy adversely influences the patient’s understanding of wound advice [6,7,15,19]. One participant in the present study felt that education could be improved because they needed to prompt the pharmacist for advice. The satisfactory patient experiences with continuity of care were associated with regular appointments, aftercare assurance and extra measures to invest time in checking on the patient’s wellbeing. Previous research examining wound care in non-pharmacy settings reported patient dissatisfaction with unpredictable booking times, lack of staffing, and inconsistency in wound care knowledge [1,8,15]. As a means of improving patient experiences and satisfaction with wound education in pharmacies, the integration of documented care plans into the wound treatment process should be considered [1]. However, this implementation of documented care plans would only be of increased value if the wound care service was to start addressing more complex and chronic wounds.

Patients felt confident in their wound care clinician’s knowledge when they had accreditation or training and conducted themselves professionally. This confidence was also seen in non-pharmacy settings when patients were aware of their practitioners’ wound care qualifications [1,4,18,20]. With patients valuing accurate wound care education and competent wound care providers, there is a case for implementing a specialized course for pharmacists to provide wound care [1]. This could help to increase the standard of wound care and reduce the variability in the quality of the service, thereby enhancing patient satisfaction [4].

When discussing improvements that can be made to pharmacy-based wound care services, patients suggested expanding wound care clinics into more communities. Patients also bemoaned the public’s lack of awareness about pharmacy-based wound care. Often, this was attributed to the inadequate promotion of these services by the stores. Moreover, pharmacists have noted when discussing the expansion of professional services, much like in pharmacy-based wound clinics, that sufficient advertising and organizational support allow these services to be noticed and succeed [16]. It can therefore be argued that advertising will be crucial in expanding the wound care service, as most patients appear to encourage and recommend it.

The expansion of the wound care service would also increase accessibility within patients’ local communities. Prior studies suggested the increased accessibility and effectiveness of wound care management came about through the expansion of service by incorporating multiple disciplines within the community [1,15]. The expansion of pharmacy-based wound care services could also be a catalyst for a more robust multidisciplinary wound care model. This could offset the financial burden on the health care system, associated with wound care in the tertiary care system, as more patients engage with pharmacy-based wound treatment in their local community.

### 4.2. Strengths and Limitations

One of the key strengths of the present study was the rigor in data analysis that came about from the independent coding of interview transcripts by the research team. This approach—along with the continuous review of emerging themes and sub-themes—ensured coding drift was minimized and improved the credibility and confirmability of the findings [14]. Another strength was that participants were from a range of different age groups and were a relatively even mix of male and female. This increases the transferability of the findings of the present study [14].

Despite the inclusion of participants with a variety of ages, we did not capture the views of younger participants, given our youngest participant was 35 years old. As such, our findings may not be transferable to younger age groups, and future research is recommended to capture the experiences of younger patients.

While we only included participants who received treatment of acute wounds, the severity and sub-types of the wounds were not captured and could influence experiences and satisfaction with the service. However, it is within the training and scope of all registered pharmacists in Australia to triage wounds’ severity and only treat minor, acute wounds [5].

A notable limitation was that participating wound care pharmacies were all based in metropolitan Perth, Western Australia and were from one particular pharmacy chain. Furthermore, the participants were recruited from pharmacies that were predominantly based in high socioeconomic areas, with most participants being recruited from one pharmacy. This limits the transferability of the results to non-metropolitan pharmacies and lower socioeconomic areas. Despite this limitation, the findings suggest a very positive outlook and experience for patients, but future studies should target lower socioeconomic areas and non-metropolitan areas to confirm the insights identified in the present study.

### 4.3. Further Research

At the time of writing, the study authors did not identify any other studies that explored patient experiences and satisfaction with acute wound care in pharmacy-based wound clinics. Given that this is the first study of its kind, the study authors recommend further investigation into the experiences and satisfaction of pharmacy-based wound care clinics in other settings, such as low socioeconomic areas, non-metropolitan areas, and countries with different health settings, to further explore the interplay between affordability, accessibility, and quality of service.

Future studies should also explore patient willingness to utilize pharmacy-based wound care services among patients who have not yet utilized the service. This would facilitate a broader understanding of potential barriers and facilitators to service uptake, thereby augmenting patient satisfaction and broader uptake of the service.

Additionally, as the present study only examined patients’ experiences with wound care services for the management of acute wounds, future research could explore whether similar experiences are found in patients who receive chronic wound care.

Finally, given that patients acknowledge the competence of pharmacists who provide wound care services (wound care pharmacist consultants), there is potential to explore the professional appetite for up-skilling more pharmacists with providing wound care services. Once there is the broader uptake of wound clinic services within community pharmacies, a survey could be appropriately conducted to estimate the true proportion of patients that are satisfied with pharmacy-based wound care clinics, as well as other considerations such as willingness to pay for the service. Findings from such a survey would serve to further improve extant wound care clinic services.

## 5. Conclusions

This is the first study to explore patient satisfaction and experiences with pharmacy-based wound clinics and outline patient insight surrounding potential improvements for the service. Participants generally had a positive outlook on the wound service provided in community pharmacies by pharmacists, noting that the accessibility and comprehensiveness of the service were of a high standard. Patients also expressed confidence in wound care pharmacist consultants who provide the service. Such positive experiences suggest a desire for the service to expand and for more pharmacists to be trained to provide wound care services. Thus, wound care services in the primary care setting have the potential to be extended beyond current service limitations to support the global wound care burden.

## Figures and Tables

**Table 1:** Summary of themes and sub-themes generated from the framework method.

Theme	Sub-Themes	Example
Accessibility of wound care services	Timely wound care service; flexibility of wound care service; location of wound care service; affordability	“Yeah, well that is a massive positive. Yeah, not having to go to a hospital, or, like, or a GP, because sometimes you have, you know, like oh my GP I have to wait a week, at least to get in to see her so, you know, I could just come here and it was a really accessible service.”
Comprehensiveness of wound care services	Adequate facilities and equipment; attention to detail; wound care education; continuity of care	“And then they had the gear, the powders and the right shape bandages for what I needed was also good.”
Confidence in wound care consultants	Adequate wound care qualifications; wound care competency; professionalism of wound care service	“…I just felt completely at rest and confident with the competency of the operator was going to give me a good result.”
Awareness and promotion of wound care services	-	“I didn’t realize there was such a thing as wound care. I didn’t realize that apart from going to your doctor… I didn’t realize there was specialized, wound care anywhere.”
Expansion of wound care services	-	“You know, I think, pharmacy generally in this space … should go all out to make sure we’ve got a wound care specialist in each location and encourage people to use the service because it’s wonderful.”

## Data Availability

Not available as we did not seek ethical approval for the release/sharing of data.

## References

[1] Monaro S., Pinkova J., Ko N., Stromsmoe N., Gullick J. (2021). Chronic Wound Care Delivery in Wound Clinics, Community Nursing and Residential Aged Care Settings: A Qualitative Analysis Using Levine’s Conservation Model. J. Clin. Nurs..

[2] Sen C.K. (2019). Human Wounds and Its Burden: An Updated Compendium of Estimates. Adv. Wound Care.

[3] Innes-Walker K., Parker C.N., Finlayson K.J., Brooks M., Young L., Morley N., Maresco-Pennisi D., Edwards H.E. (2019). Improving Patient Outcomes by Coaching Primary Health General Practitioners and Practice Nurses in Evidence Based Wound Management at on-Site Wound Clinics. Collegian.

[4] Norman R.E., Gibb M., Dyer A., Prentice J., Yelland S., Cheng Q., Lazzarini P.A., Carville K., Innes-Walker K., Finlayson K. (2016). Improved Wound Management at Lower Cost: A Sensible Goal for Australia: Improved Wound Management in Australia. Int. Wound J..

[5] Pharmaceutical Society of Australia (2017). Professional Practice Standards—Version 5—June 2017.

[6] Dhar A., Needham J., Gibb M., Coyne E. (2020). The Outcomes and Experience of People Receiving Community-based Nurse-led Wound Care: A Systematic Review. J. Clin. Nurs..

[7] Fearns N., Heller-Murphy S., Kelly J., Harbour J. (2017). Placing the Patient at the Centre of Chronic Wound Care: A Qualitative Evidence Synthesis. J. Tissue Viability.

[8] Squitieri L., Tsangaris E., Klassen A.F., Haren E.L.W.G., Poulsen L., Longmire N.M., Alphen T.C., Hoogbergen M.M., Sorensen J.A., Cross K. (2020). Patient-reported Experience Measures Are Essential to Improving Quality of Care for Chronic Wounds: An International Qualitative Study. Int. Wound J..

[9] Welsh L. (2018). Wound Care Evidence, Knowledge and Education Amongst Nurses: A Semi-Systematic Literature Review. Int. Wound J..

[10] Given L.M. (2008). Semi-Structured Interview. The SAGE Encyclopedia of Qualitative Research Methods.

[11] Dubhashi S.P., Sindwani R.D. (2015). A Comparative Study of Honey and Phenytoin Dressings for Chronic Wounds. Indian J. Surg..

[12] Mason M. (2010). Sample Size and Saturation in PhD Studies Using Qualitative Interviews. Forum Qual. Soz. Forum Qual. Soc. Res..

[13] Gale N.K., Heath G., Cameron E., Rashid S., Redwood S. (2013). Using the Framework Method for the Analysis of Qualitative Data in Multi-Disciplinary Health Research. BMC Med. Res. Methodol..

[14] Lincoln Y.S., Guba E.G. (1985). Naturalistic Inquiry.

[15] McCaughan D., Sheard L., Cullum N., Dumville J., Chetter I. (2018). Patients’ Perceptions and Experiences of Living with a Surgical Wound Healing by Secondary Intention: A Qualitative Study. Int. J. Nurs. Stud..

[16] Hattingh L., Sim T.F., Sunderland B., Czarniak P. (2020). Successful Implementation and Provision of Enhanced and Extended Pharmacy Services. Res. Soc. Adm. Pharm..

[17] Goh L.J., Zhu X. (2018). Exploring Patient and Caregiver Perceptions of Primary Healthcare Sector Home Care for Simple Acute Wounds. Adv. Ski. Wound Care.

[18] Wang S.C., Anderson J.A., Jones D.V., Evans R. (2016). Patient Perception of Wound Photography: Patient Perception of Wound Photography. Int. Wound J..

[19] Weller C., Richards C., Turnour L., Green S., Team V. (2020). Vascular Assessment in Venous Leg Ulcer Diagnostics and Management in Australian Primary Care: Clinician Experiences. J. Tissue Viability.

[20] Moffatt C., Murray S., Keeley V., Aubeeluck A. (2017). Non-Adherence to Treatment of Chronic Wounds: Patient Versus Professional Perspectives: Non-Adherence in Chronic Wounds. Int. Wound J..

